# Hydrogen Photoproduction by *Rhodopseudomonas palustris* 42OL Cultured at High Irradiance under a Semicontinuous Regime

**DOI:** 10.1155/2012/590693

**Published:** 2012-07-15

**Authors:** Pietro Carlozzi

**Affiliations:** Istituto per lo Studio degli Ecosistemi, Sede di Firenze, Consiglio Nazionale delle Ricerche, Polo Scientifico, Via Madonna del Piano n. 10, Sesto Fiorentino, 50019 Firenze, Italy

## Abstract

The main goal of this study was to increase the hydrogen production rate improving the culture technique and the photobioreactor performances. Experiments were carried out at a constant culture temperature of 30°C and at an average irradiance of 480 W m^−2^ using a cylindrical photobioreactor (4.0 cm, internal diameter). The culture technique, namely, the semicontinuous regime for growing *Rhodopseudomonas palustris* 42OL made it possible to achieve a very high daily hydrogen production rate of 594 ± 61 mL (H_2_) L^−1^ d^−1^. This value, never reported for this strain, corresponds to about 25 mL (H_2_) L^−1^ h^−1^, and it was obtained when the hydraulic retention time (HRT) was of 225 hours. Under the same growth conditions, a very high biomass production rate (496 ± 45 mg (dw) L^−1^ d^−1^) was also achieved. Higher or lower HRTs caused a reduction in both the hydrogen and the biomass production rates. The malic-acid removal efficiency (MA_re_) was always higher than 90%. The maximal hydrogen yield was 3.03 mol H_2_ mol MA^−1^ at the HRT of 360 hours. The highest total energy conversion efficiency was achieved at the HRT of 225 hours.

## 1. Introduction

Hydrogen has been recognized as a promising energy carrier of the future because it is clean, recyclable, and efficient [1]. Biohydrogen technologies are still in their infancy. Existing technologies offer potential for practical applications, but if biohydrogen systems are to become commercially competitive, they must be able to synthesize hydrogen at rates that are sufficient to power fuel cells of a sufficient size to carry out practical work [2]. Purple nonsulfur photosynthetic bacteria can decompose organic acids by using light energy and nitrogenase in a photofermentation process [3]. *Rhodopseudomonas palustris* WP3-5 was used to produce hydrogen phototrophically from acetate and butyrate, which are the major soluble products from acidogenic dark fermentation [4]. The organic carbon source used for this study was malic acid (MA), which is a compound of wine-distillery waste [5]. An efficient biological hydrogen production process that uses cheaper materials would undoubtedly make the system more competitive with the conventional hydrogen generation process in the future [6]. Nevertheless, high hydrogen yield (*Y*_H_) remains to be the ultimate goal and challenge for the biohydrogen research and development [7]. The key points for improving the *Y*_H_ are also advancements in hydrodynamic aspects, bioreactor design, gas separation, light intensity and its distribution inside culture thickness [8].

Over the years, many scientists investigated the photofermentation process using nonsulfur photosynthetic bacteria for indoor hydrogen photoproduction under batch growth conditions [9–11]. Recently some papers described different growth strategies for enhancing the photohydrogen production yield: (i) fed-batch operation [12]; (ii) repeated fed-batch cultures [13]; (iii) fill and draw (F/D) operations; (iv) continuous culture [14]. Three of the above strategies (batch, F/D, and continuous culture) were compared by Chen et al. [14]. They enhanced the H_2_ production rate till 38.2 mL L^−1^ h^−1^. The F/D strategy is similar to the semicontinuous regime (SCR) used by algologists to grow outdoors cyanobacteria or microalgae [15–17]. F/D is a regime in which the dilution rate is the fixed parameter and the biomass concentration is the outcome. The SCR permits working (outdoors) at a prefixed biomass concentration. At sunset, an appropriate culture volume is withdrawn from the reactor and replaced with the same volume of fresh medium, so as to maintain the fixed biomass concentration [18]. This technique used outdoors is very important when a pre-fixed biomass concentration is required, in order to optimize the biomass output rate or a specific production of natural biomolecules of peculiar interest. During the SCR, the dilution rate is expressed in day^−1^, without reaching a steady state (i.e., unstable cell concentration), while under a continuous regime the dilution rate is expressed in h^−1^, and a very steady state is reached (i.e., stable value of cell concentration). When the investigation is carried out indoors and at a fixed light intensity, it is better to work using a pre-fixed dilution rate to reach a semisteady state condition, under which the biomass concentration follows an up and down pattern. Theoretically, the F/D operation is a suitable strategy for a slow-growing microorganism like *R. palustris* to obtain a long-term steady activity for photohydrogen production [14]. In F/D operations carried out by Chen et al. [14], half of the culture medium (400 mL) was discharged every 24 hours, and the same volume of fresh medium was then rapidly fed into the reactor to reach a final volume of 800 mL. The discharge time and the quantity of replacement in the F/D operation were designed to imitate an average hydraulic retention time (HRT_avg_) of 48 hours. 

Other relevant questions debated in the scientific community concern culture thickness, light path, and mixing to expose cells to certain light/dark low-frequency cycles [19]. The light path should be about 1/10 of the culture thickness [20]. In 2008, culturing *R. palustris* at 483 W m^−2^, we demonstrated that the hydrogen production rate has an inverse relationship with the culture thickness because the higher the hydrogen production rate, the lower the culture thickness [21]. In 2009, we studied the effect of irradiance on the hydrogen production rate based on culture volume (HPR_*V*_) under fed-batch operations attaining the maximum HPR_*V*_ of 17.22 mL L^−1^ h^−1^ at 500 W m^−2^ [22]. In view of the relevant results obtained in 2008 and 2009 by our group [21, 22], in the present study, we used a cylindrical photobioreactor to investigate on hydrogen photoproduction at high irradiance (480 W m^−2^), using a SCR as growth strategy. On the basis of the previous investigation [22], where the higher the HPR_*V*_ the lower the light conversion efficiency, the main goal of this investigation was the improvement of the HPR_*V*_ together with the biomass output rate, even if the high irradiance of 480 W m^−2^ could impose a penalty to the light conversion efficiency. All these three relevant aspects were discussed in the present study.

## 2. Materials and Methods

**Description of the Photobioreactor**

**Figure 1:**
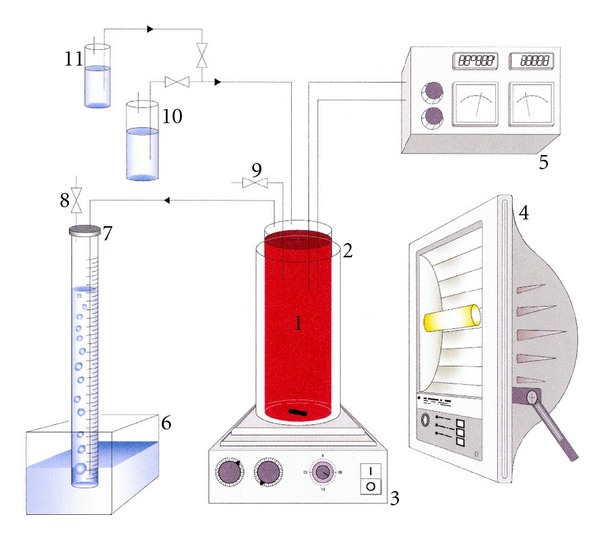
Schematic diagram of the cultural system. (1) Heat exchanger water bath; (2) photobioreactor; (3) magnetic stirrer; (4) lamp; (5) control unit; (6) saline solution basin; (7) graduated column trap; (8) gas sampling; (9) culture sampling; (10) MA stock solution; (11) glutamic acid stock solution.

The photobioreactor used for the production of photobiological hydrogen by means of a photofermentative process was a cylindrical glass photobioreactor (internal diameter (i.d.): 4.0 cm; working volume: 250 mL) placed in a heat exchanger-Plexiglas water bath at a constant temperature; the culture was mixed using a magnetic stirrer (Figure 1). All experiments were carried out in a thermostatic room and under atmospheric pressure. The gas produced by bacteria cells was first made to flow into a basin containing a CO_2_-absorber (saline solution of NaOH); the hydrogen was then trapped in a calibrated column, where it was collected, and the volume was measured to determine the hydrogen production [8]. The calibrated column was refilled with a saline solution of NaOH every morning.

### 2.1. Microorganism and Culture Conditions

*R. palustris* 42OL was precultured, at a constant temperature of 30 ± 0.2°C, in anaerobic condition (Sovirel bottles, 250 mL) under continuous light of 480 W m^−2^ using the following medium composition (1.0-litre volume): 1.63 g C_4_H_6_O_5_, 0.5 g NH_4_Cl, 1.0 g KH_2_PO_4_; 0.4 g NaCl, 0.4 g MgSO_4_·7H_2_O, 0.05 g CaCl_2_·2H_2_O, 0.1 mg p-aminobenzoic acid, and 10 mL of mineral solution for micronutrients. Mineral solution (1.0 L) contained 1.0 mg CuCl_2_·2H_2_O, 2.0 mg NiCl_2_·6H_2_O, 3.0 mg MnCl_2_·4H_2_O, 10 mg ZnSO_4_·7H_2_O, 20 mg CoCl_2_·6H_2_O, 30 mg H_3_BO_3_, 200 mg FeSO_4_·7H_2_O, and 500 mg Na_2_MoO_4_·7H_2_O. MA (C_4_H_6_O_5_) was neutralized with NaOH salt and the pH of the medium was adjusted to 6.8 by using 1 M HCl or NaOH solutions. For hydrogen photoproduction, the growth medium was modified, the carbon source (C_4_H_6_O_5_) concentration was increased to 3.26 g L^−1^, and the nitrogen source (NH_4_Cl) was replaced with 1.0 g C_5_H_8_NNaO_4_ [23]. All experiments were carried out using a Halogen lamp (250-W OSRAM power-star HQI-TS), under continuous light at 480 W m^−2^ and at a constant temperature of 30 ± 0.2°C. The cultures were operated according to the SCR as growth strategy applied after an initial start-up phase (fed-batch growth) described in the previous paper [22]. The SCR was applied after reaching a suitable Bchl concentration (≥20 mg L^−1^). Every 72 hours, the appropriate culture volumes were withdrawn from the reactor and replaced with an equal volume of fresh medium to imitate three different HRT_avg_ (360, 225, and 138 hours). The HRT_avg_ = volume/average flow rate was determined according to Chen et al. [14]. Moreover, culture replacement was accomplished each time (culture time of 72 hours) adding to the culture: MA (3.26 g L^−1^) and glutamate (1.0 g L^−1^). In this way, *R. palustris* 42OL was spared any unsuitable situation that might have halted the growth for lack of macronutrients, such as C and N. This feeding strategy was used for long-term investigation (>720 hours excluding the start-up phase), until a semisteady state condition was reached, and data were collected. The semisteady state condition consists in a repetitive cycle were a stable up and down of dry-weight biomass concentration was achieved. Each cycle had a cultivation time of 72 hours.

### 2.2. Analytical Methods

To determine dry-weight biomass, 5 mL of culture were diluted with distilled water to 50 mL and were then filtered, without compact cells, through a preweighed cellulose nitrate membrane that had a 0.45 *μ*m pore size (Sartorius GmbH, 3400 Göttingen, Germany). Moreover, the prewashed sample was suspended again in 50 mL of distilled water, rapidly filtered, and dried at 105°C until a constant weight was reached [18]. Bacteriochlorophyll (Bchl) was determined in accordance with [23]. Cultures were irradiated with a 250 W OSRAM power-star HQI-TS lamp. The irradiance was measured using a Quantum/Radiometer/Photometer (model LI-185B, LI-COR, Lincoln, NE, USA). In order to determine organic-acid concentrations in the bacteria cultures, a HPLC (Thermo Finnigan-Spectra System 6000 LP) was utilised. The HPLC was equipped with a C18 analytical column (250 × 4.6 mm), and the column temperature was 25°C. After disposable syringe filter units (MFS-13 mm, 0.45 *μ*m pore size) were used to remove the cells, the supernatant was tested for MA. The mobile phase was a solution of water + 0.1% H_3_PO_4_, and the flow was 1.0 mL min^−1^. The gas produced (after removing the CO_2_ with a saline solution of NaOH) was trapped in a calibrated column, where it was collected and the volume was measured to determine hydrogen production. No CO_2_ was found inside the calibrated column. This was controlled by analysis using a Perkin-Elmer Autosystem gas chromatograph equipped with a TCD detector and a Silica Gel 60/80 Grade 12 column (Alltech, Deerfield). The carrier gas was helium; known amounts of pure gases were used to calibrate the instrument [8]. Elemental analyses of the biomasses (C-H-N-O) were performed in triplicate, according to Carlozzi et al. [23], using an elemental analyzer (model 1106, Carlo Erba Instrumentation, Milan).

### 2.3. Light Conversion Efficiency

Light conversion efficiency (*η*) was determined by using the following equation:

(1) η  (%)=33.61ρH2VH2IAtH2×100.

#### 2.3.1. Total Energy Conversion Efficiency

The total energy conversion efficiency (*η*′) was determined according to Carlozzi [22] using the following equation:

(2) η′  (%)=33.61ρH2VH2+(PB(−ΔPB))IAtH2+(MAc(−ΔMA))+(GAc(−ΔGA))×100,

where *ρ*H_2_ is the density of hydrogen (gas) (g L^−1^); *V*H_2_ is the volume of H_2_ produced (L); *P*_B_ is the total ash-free dry biomass produced (g); (−Δ*P*_B_) is the heat of combustion of ash-free biomass (dw) (kcal g^−1^); *I* is the irradiance (W m^−2^); *A* is the irradiated area of the photobioreactor (m^−2^), which was calculated as being half of the cylindrical reactor surface (2*πr*_*i*_*h*), with *r*_*i*_ and *h* indicating, respectively, the internal radius and the height of the cylindrical reactor; *t*H_2_ is the hydrogen evolution time (hours); MA_c_ is the malic acid consumed (g); (−ΔMA) is the heat of combustion of the MA (kcal g^−1^); GA_c_ is the glutamic acid consumed (g); (−ΔGA) is the heat of combustion of GA (kcal g^−1^).

## 3. Results

**Figure 2:**
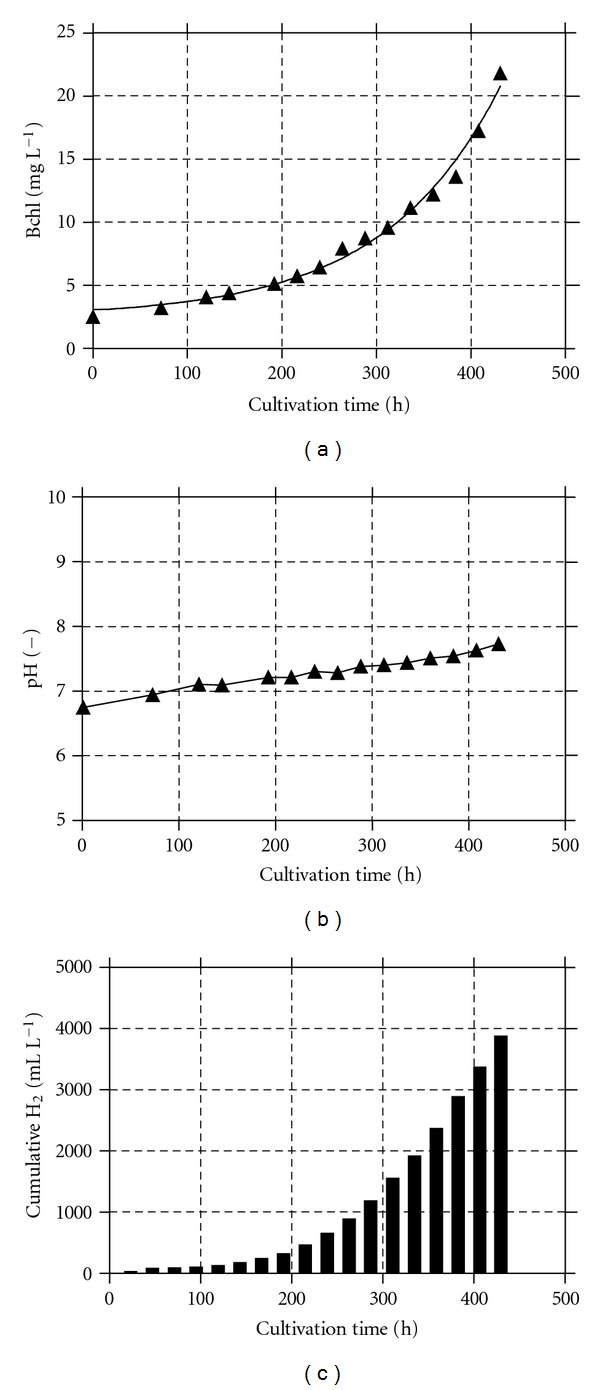
Start-up phase of *R. palustris* 42OL grown at 30°C with an irradiance of 480 W m^−2^. (a) Changes in the Bchl concentration, (b) pH, and (c) cumulative H_2_ versus cultivation time.

**Figure 3:**
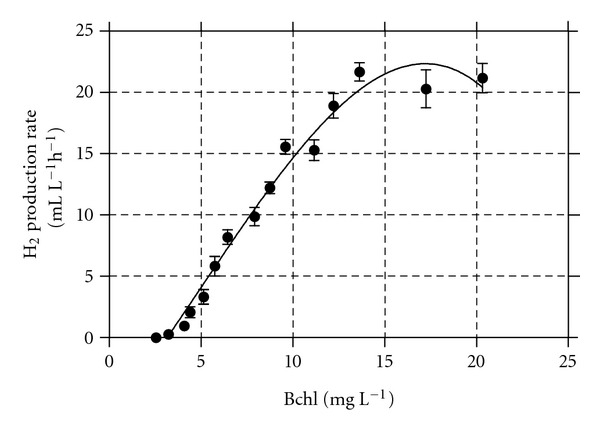
Hydrogen production rate versus the Bchl concentration attained under batch growth conditions, at 30°C with an irradiance of 480 W m^−2^.

**Figure 4:**
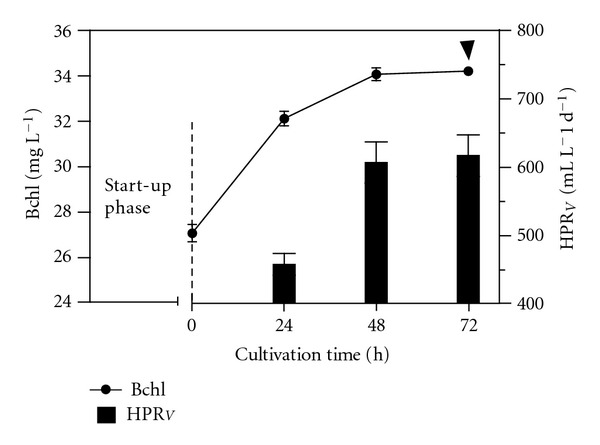
Changes in the Bchl concentration versus the cultivation time and relative HPR_*V*_ achieved in time, under SCR, at 30°C with an irradiance of 480 W m^−2^. The HRT was 360 hours. The arrow indicates the cultivation time when we withdrew 50 mL of culture volume from the reactor and replaced with an equal volume of fresh medium.

*R. palustris* culture was operated under fed-batch growth conditions (start-up phase) before applying the SCR as growth strategy; the results are shown in Figure 2. Over a cultivation time of more than 430 hours, the Bchl concentration reached 21.8 mg L^−1^, the pH grew to 7.75, and the cumulative hydrogen was about 4.0 litres. The HPR_*V*_ was also checked. The exploration made it possible to discover the relationship between the HPR_*V*_ and the Bchl concentration. The results are shown in Figure 3. The investigation, succeeded in establishing, roughly, the point at which the HPR_*V*_ reached its upper limit as a function of the Bchl concentration. Starting from this point, the growth strategy was changed from the fed-batch to the SCR. Results attained at the HRT of 360 hours are shown in Figure 4. The SCR growth strategy caused a stable up and down Bchl pattern (27.22 ± 0.42 mg L^−1^ to 34.22 ± 0.23 mg L^−1^), which was assumed as semisteady state conditions; data were collected under this latter condition. The daily HPR_*V*_ increased in accordance with the cultivation time reaching the maximum (619 ± 53 mL (H_2_) L^−1^ d^−1^) at 72 hours.

**Figure 5:**
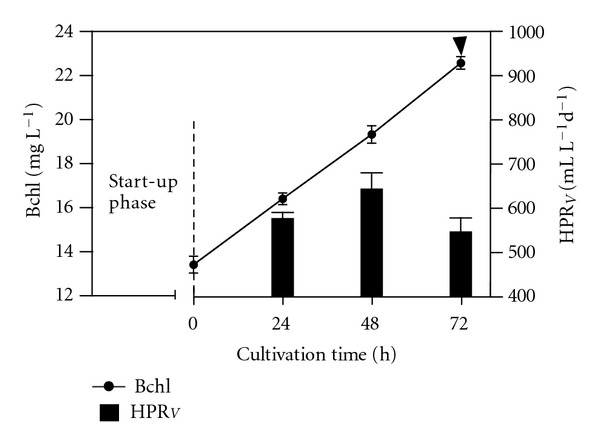
Changes in the Bchl concentration versus the cultivation time and relative HPR_*V*_ achieved in time, under SCR, at 30°C with an irradiance of 480 W m^−2^. The HRT was 225 hours. The arrow indicates the cultivation time when we withdrew 80 mL of culture volume from the reactor and replaced with an equal volume of fresh medium.

**Figure 6:**
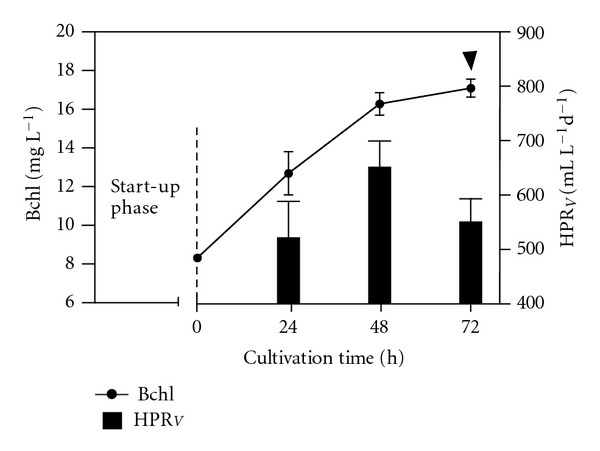
Changes in the Bchl concentration versus the cultivation time and relative HPR_*V*_ achieved in time, under SCR, at 30°C with an irradiance of 480 W m^−2^. The HRT was 138 hours. The arrow indicates the cultivation time when we withdrew 120 mL of culture volume from the reactor and replaced with an equal volume of fresh medium.

In order to further increase the HPR_*V*_, we tested two other HRTs (225 hours and 138 hours). The results are shown, respectively, in Figures 5 and 6. As theoretically expected, the Bchl concentrations decreased in accordance with the HRT; therefore, the shorter the HRT, the lower the Bchl concentration.

Obviously, the up and down pattern of the Bchl remained, but it was expressed at lower levels: at HRT = 225 hours (13.42 ± 0.66 mg L^−1^ to 22.6 ± 0.49 mg L^−1^) in Figure 5; at HRT = 138 hours (8.34 ± 0.21 mg L^−1^ to 17.15 ± 0.82 mg L^−1^) in Figure 6. On the contrary, the daily HPR_*V*_ reached 649 ± 59 mL (H_2_) L^−1^ d^−1^, at the cultivation time of 48 hours, when the HRT was 225 hours (Figure 5). The daily HPR_*V*_ reached its top value (655 ± 85 mL (H_2_) L^−1^ d^−1^) at the same cultivation time of 48 hours, when the HRT was 138 hours (Figure 6). The corresponding hourly average HPR_*V*_ was 27.3 ± 3.5 mL (H_2_) L^−1^ h^−1^.

Table 1 provides a summary of the results attained during the three HRTs. All data were collected once the semisteady state conditions were reached. Both the highest HPR_*V*_ and productivity were achieved at the HRT of 225 hours. The MA_re_ showed an inverse relationship with the HRT: the higher the HRT, the lower the MA_re_. The investigation into the yields is also shown in Table 1. The dry biomass, produced per malic acid consumed (*Y*_B_) expressed as carbon equivalent (C_Eq_), reached the highest value (0.451) at the HRT of 225 hours. The *Y*_H_ was 3.03 mol H_2_ mol MA^−1^ at HRT = 360 hours and decreased at shorter HRTs. Since the theoretical *Y*_H_ for malic acid is 6.0 mol H_2_ mol MA^−1^, the average *Y*_H_ value we attained experimentally was about 50% of the theoretical one.

Equations (1) and (2) were used in order to calculate both *η* and *η*′ achieved under the SCR of growth strategy. The elemental composition of ash-free dry biomass was checked to determine the heat of combustion (−Δ*P*_B_) of all the three biomasses harvested (HRT = 138 hours; 225 hours; 360 hours). The results are shown in Table 2. The highest *η* and *η*′ (0.31% and 0.78%, resp.) were achieved at the optimal HRT of 225 hours. Both conversion efficiencies decreased when cultures were operated at suboptimal HRTs.

## 4. Discussion

In 2009, Ren et al. [13] enhanced photohydrogen production yield using a repeated fed-batch cultures; they attained an average *Y*_H_ of 3.17 mol H_2_/mol acetate culturing *R. faecalis*, RLD-53. In the same year, we demonstrated that the fed-batch growth is a promising growth strategy to produce biohydrogen [12]. The plan we used, in the present study, to improve the hydrogen production rate, was a suitable growth strategy (SCR) similar to the F/D operation suggested in 2006 by Chen et al. [14]. Since under fed-batch growth conditions the effect of irradiance growing on hydrogen photoproduction demonstrated that a high irradiance (500 W m^−2^) was adequate to achieve the maximal HPR_*V*_ of 17.22 mL H_2_ L^−1^ h^−1^ [22], to test *R. palustris* 42OL under the SCR, we irradiated the photobioreactor with the irradiance of 480 W m^−2^.

The relationship of HPR_*V*_ versus Bchl concentration showed that the optimal Bchl was about 1.5 times higher than that we found in 2009 [12]. At the HRT of 225 hours, the average hydrogen production rate (HP_*A*_) was 594 ± 61 mL (H_2_) L^−1^ d^−1^, which is 2.7 times higher than the highest value we had previously reported (222 mL (H_2_) L^−1^ d^−1^) using a cylindrical photobioreactor (i.d. 7.6 cm) [21]. Moreover, it is worth noting that the HP_*A*_ of 594 ± 61 mL (H_2_) L^−1^ d^−1^, obtained under semisteady state condition, corresponds to 24.75 mL (H_2_) L^−1^ h^−1^, which can be considered to be the highest average value ever reported for *R. palustris* 42OL and among the highest values reported for several different *R. palustris* strains [4, 11–14]. In this study using a halogen lamp, we irradiated the photobioreactor at 480 W m^−2^ to investigate the SCR growth strategy and the HPR_*V*_ of *R. palustris* 42OL enhanced to 25 mL H_2_ L^−1^ h^−1^. Irradiating *R. palustris* WP 3-5 with the same halogen lamp, at 95 W m^−2^, Chen et al. [14] achieved 20.9 mL H_2_ L^−1^ h^−1^, which increased to 38.2 mL H_2_ L^−1^ h^−1^, when a ternary-light-source system was used, demonstrating the high relevance of the light quality source on hydrogen photoproduction. We did not attain any benefit by increasing the HRT further (Table 1). At the HRT of 225 hours, the biomass production rate was maximal (496 ± 04 mg (dw) L^−1^ d^−1^) and reduced, respectively, of 1.9 and 1.3 times when the HRT was of 360 and 138 hours. Even if biomass production is in competition with hydrogen photoproduction, *R. palustris* 42OL is able to use volatile acids as carbon source for growth and the concomitant photobiological evolution of hydrogen [8]. The biomass production rate could assume a very high significance if we consider the potential of the two green energy sources (bioH_2_ and biodiesel) since *R. palustris* 42OL is an oleaginous bacteria [8]. Microorganisms that can accumulate lipids at more than 20% of their dry biomass are defined as oleaginous species [24]. We did not investigate about this topic, but we would like to remark that what could be lost as hydrogen could be stored as biomass rich in oil. 

The highest *η*′ value obtained under the SCR growth strategy was compared with the one reported in a previous paper, in which experiments were carried out under fed-batch growth conditions [22]. It was noted that the *η*′ value achieved in this study, at the HRT of 225 hours, was 13% higher than the one obtained previously when the irradiance was 500 W m^−2^ and the average hydrogen production rate improved of 44% if compared with the previously attained value (17.21 mL L^−1^ h^−1^) [22]. The remarkable improvement in the average hydrogen production rate attained in this study was attributed to the SCR together with the suitable culture thickness attained thanks to the 4.0 cm i.d. of the photobioreactor. The last is fully in agreement with what we demonstrated elsewhere [21]. On the contrary, the total light conversion efficiency remained moderate, above all because the light intensity was high (480 W m^−2^). In 2006 Chen et al. [14] reported, under F/D operation, a light conversion efficiency of 1.93% using optical-fiber-assisted illumination systems (light intensity = 95 W m^−2^) and acetate as a carbon substrate for producing hydrogen by means of *R. palustris* WP3-5. The major drawback for optical fiber-solar light has been the instability of the solar energy supplied, which is a general problem that limits the applicability and productivity of outdoor photobioreactors. In 2010, Liao et al. [25] reported a light conversion efficiency of 8.9% using LEDs (wavelength: 590 nm) to illuminate *R. palustris* CQK 01, using glucose as a substrate. Combining optical fiber-solar light with a multi-LED light source and other artificial light sources have also been suggested by Chen et al. [26]. Our results regarding both the light conversion efficiency (0.31%) and the total energy conversion efficiency (0.78%) if compared with the ones reported above [25] show notable differences. Even if glucose was used as a substrate and single-wavelength LED lamp (590 nm) was employed to irradiate the photobioreactor at 5000 Lux by Liao et al. [25], our results provide an unfavourable comparison, but they can be considered very good because they were obtained under high light intensity of 480 W m^−2^ which is not very often investigated, even if it is relevant because it can be found outdoors. To overcome the low light conversion efficiency achievable at high irradiance, two different strategies can be used: (i) the solar rays condensed using Fresnel lenses and transmitted by means of optical fibres for photobioreactor assistance and (ii) the dilution of solar radiation through culture lamination in a multirow photobioreactor [27]. The first is a high technology, while the second is a rather unrefined technology that is simple and practical. We consider that the debate on this is still open. The MA_re_ was always higher than 90%. We believe it to be a meaningful aspect because the effluent outlet contains only very little residual substrate concentrations, which is especially interesting in setting up a process for both hydrogen photoproduction together with wastewater purify treatment, that is, using wine distillery waste materials as feeding because it contains the organic substance as MA [5]. 

## 5. Conclusions 

In this study, we have demonstrated that the hydrogen production rate achievable by means of a photofermentative process using purple nonsulfur photosynthetic bacteria such as *R. palustris* 42OL can be increased significantly (about twice) by growing it: (i) in a short i.d. of cylindrical photobioreactor, under SCR as growth strategy and (ii) at high irradiance of 480 W m^−2^, similar to the outdoor conditions that bacteria cells will experience if they grow under natural solar radiation. The experimental laboratory conditions under which all our experiments were carried out demonstrated that reactor performances could be improved significantly if the adequate SCR was established. Generally speaking, the gap of both *η* and *η*′ achievable at high irradiance as compared with the one attainable at low irradiance is remarkable [22]. As we demonstrated elsewhere [27], a light dilution could attain a notable effect on the conversion efficiency under excessive irradiance conditions.

## Figures and Tables

**Table 1:** Results achieved under continuous irradiance (480 W m^−2^) at selected HRTs.

HRT (hours)	HPR_*V*_ (mL L^−1^ d^−1^)	Productivity (g (dw) L^−1^ d^−1^)	MA_re_ (%)	Yields	
				*Y*_B_ (C_Eq_B C_Eq_ MA_c_^−1^)	*Y*_H_* (mol H_2_ mol MA^−1^)
360	563 ± 87	0.254 ± 0.011	90.1	0.248	3.03
225	594 ± 61	0.496 ± 0.045	93.7	0.451	2.99
138	578 ± 99	0.373 ± 0.015	99.7	0.307	2.63

^∗^The theoretical *Y*_H_ is 6.0 mol H_2_ mol MA^−1^.

**Table 2:** Elemental biomass composition and heat of combustion of ash-free biomasses of *R. palustris* 42OL grown under SCR, at three different HRTs. The conversion efficiencies (*η* and *η*′) have been reported as well. Cells were harvested when the semisteady state condition was reached.

Ash-free biomass composition^∗^			
		HRT (hours)	
	360	225	138
C (%)	55.30 ± 0.21	54.95 ± 0.57	55.07 ± 0.46
H (%)	8.52 ± 0.11	8.45 ± 0.29	8.51 ± 0.15
N (%)	8.31 ± 0.03	8.36 ± 0.09	8.37 ± 0.10
O (%)	27.87 ± 0.49	28.24 ± 0.31	28.05 ± 0.22
−Δ*P*_B_ (Kcal g (dw)^−1^)	6.330	6.268	6.301
*η* (%)	0.29	0.31	0.30
*η*′ (%)	0.54	0.78	0.66

^∗^Analyses were performed in triplicate, mean values ± SD are reported.

## Abbreviations

*A*: Irradiated area of the photobioreactor (m^−2^)
C_Eq_: Carbon equivalent (—)
F/D: Fill and draw operations
GA: Glutamic acid
GA_c_: Glutamic acid consumed (g)
*h*: Height of the cylindrical reactor (m^−2^)
HP_*A*_: Average hydrogen production rate (mL H_2_ L^−1^ d^−1^)
HPR_*V*_: Hydrogen production rate based on culture volume (mL H_2_ L^−1^ h^−1^)
HRT: Hydraulic retention time (hours)
HRT_avg_: Average hydraulic retention time (hours)
*I*: Irradiance (W m^−2^)
MA: Malic acid
MA_c_: Malic acid consumed (g)
MA_re_: Malic acid removal efficiency (%)
P_B_: Ash-free biomass productivity (g)
*r*_*i*_: Internal radius of the cylindrical reactor (m)
SCR: Semicontinuous regime
*t*H_2_: Hydrogen evolution time (hours)
*V*H_2_: Volume of hydrogen produced (L)
*Y*_B_: Carbon equivalent of dry biomass produced per malic acid consumed (C_Eq_BC_Eq_MA_c_^−1^)
*Y*_H_: Hydrogen yield (mol H_2_ mol MA^−1^).

### Greek Symbols

(−ΔGA): Heat of combustion of GA (Kcal g^−1^)
(−ΔMA): Heat of combustion of MA (Kcal g^−1^)
(−Δ*P*_B_): Heat of combustion of ash-free dry-biomass (Kcal g^−1^)
*η*: Light conversion efficiency (%)
*η*′: Total energy conversion efficiency (%)
*ρ*H_2_: Density of hydrogen (gas) (g L^−1^).
